# Prediction of Milk Consumption Among Iranian Pregnant Women: Application of the Theory of Planned Behavior

**DOI:** 10.5812/ircmj.1912

**Published:** 2013-05-05

**Authors:** Tahereh Dehdari, Fatemeh Manafi, Amal Saki

**Affiliations:** 1 Department of Health Education and Promotion, Faculty of Health, Tehran University of Medical Sciences, Tehran, IR Iran; 2 Faculty of Health, Tehran University of Medical Science, Tehran, IR Iran; 3 Department of Biostatistics, Faculty of Medical Science, Tarbiat Modares University, Tehran, IR Iran

**Keywords:** Milk, Human, Pregnancy, Behavior

## Dear Editor,

Studies showed that Iranian pregnant women did not take enough calcium and dairy products such as milk (1). Adequate milk consumption is crucial during pregnancy to meet calcium needs (2). Various factors such as attitudes, beliefs, habit, sensory and expectations are determinants of the consumption of milk (3). The Theory of Planned Behavior (TPB) has received much attention in the health domain and has been previously shown to successfully predict food hygiene behavior (3). According to this theory, the instantaneous predictor of behavior is the intention that is itself predicted by attitude, subjective norms, and perceived behavioral control (4). In this descriptive-analytical study, a convenience sample included 112 pregnant women referred to the urban health centers of Tehran University of Medical sciences, Tehran, Iran were selected and filled out questionnaires. Findings demonstrated that high proportion of pregnant women did not take enough milk. The mean milk consumption was 1.8 cups per day. This Finding is consistent with Park and Ureda (2). They reported that the mean of milk consumption was 1.8 cups per day among pregnant women in South California. Also, Karandish et al. (1) reported that mean daily intakes of dairy products in pregnant women in Ahwaz city was 1.3 servings per day. It is necessary to be more considerate to milk consumption in pregnancy. The intention to milk consumption significantly predicted daily milk consumption among subjects. On the other hand, by increasing behavioral intention milk consumption increased as well. Kim et al. (5) found that intention to consumption of dairy significantly explained dairy consumption in older adults. The attitude and perceived behavioral control constructs significantly predicted the intention to milk consumption in Iranian pregnant women whereas subjective norms did not. These data affirm that subjective norms had no significant effect on the intention toward milk consumption among this group. Findings of such studies lend support of this finding (6, 7). Raats et al. (8) demonstrated that in such studies subjective norms had independent effects on intention and in contrast to studies where subjective norms had no effect. They expressed that according to Ajzen and Fishbein comment, perceived social pressure to perform or not to perform a behavior may not be adequately assessed by their subjective norm measure. The attitudes represent the overall evaluations of the behavior as positive or negative among individuals and perceived behavioral control is the individual’s perception of the extent to which performance of the behavior is easy or difficult (9). Present study indicated that by increasing perceived behavioral control and attitude in participants milk consumption increased. As a result, assessment of behavioral intention of milk consumption of pregnant women is crucial for health care practitioners. Also, for increasing behavioral intention toward milk consumption and designing effective interventions it is better to focus on attitude and perceived behavioral control of pregnant women.

## References

[1] Karandish Majid, Mohammadpour-Ahranjani Behnoush, Rashidi Arash, Maddah Mohsen, Vafa Mohammad-Reza, Neyestani Tirang-Reza (2005). Inadequate intake of calcium and dairy products among pregnant women in Ahwaz City, Iran.. Mal J Nutr..

[2] Park Kyungwon, Ureda JohnR (1999). Specific motivations of milk consumption among pregnant women enrolled in or eligible for WIC.. J Nut Edu..

[3] Saba A, Moneta E, Nardo N, Sinesio F (1998). Attitudes, habit, sensory and liking expectation as determinants of the consumption of milk.. Food Qual Prefer..

[4] Zhang J, Shi L, Chen D, Wang J, Wang Y (2009). Using the theory of planned behavior to examine effectiveness of an educational intervention on infant feeding in China.. Prev Med..

[5] Kim K, Reicks M, Sjoberg S (2003). Applying the theory of planned behavior to predict dairy product consumption by older adults.. J Nutr Educ Behav..

[6] Brewer JL, Blake AJ, Rankin SA, Douglass LW (1999). Theory of Reasoned Action predicts milk consumption in women.. J Am Diet Assoc..

[7] Berg C, Jonsson I, Conner M (2000). Understanding choice of milk and bread for breakfast among Swedish children aged 11-15 years: an application of the Theory of Planned Behaviour.. Appetite..

[8] Raats MM, Shepherd R, Sparks P (1993). Attitudes, obligations and perceived control: predicting milk selection.. Appetite..

[9] Ajzen I (1991). The theory of planned behavior.. Organ Behav Hum Decis Process..

